# Galectin-9 protects humanized-ACE2 immunocompetent mice from SARS-CoV-2 infection

**DOI:** 10.3389/fimmu.2022.1011185

**Published:** 2022-10-17

**Authors:** Stephen T. Yeung, Thomas A. Premeaux, Li Du, Toshiro Niki, Satish K. Pillai, Kamal M. Khanna, Lishomwa C. Ndhlovu

**Affiliations:** ^1^ Division of Infectious Diseases, Department of Medicine, Weill Cornell Medicine, New York, NY, United States; ^2^ Vitalant Research Institute, San Francisco, CA, United States; ^3^ Department of Laboratory Medicine, University of California San Francisco, San Francisco, CA, United States; ^4^ Departments of Immunology and Immunopathology, Kagawa University, Kagawa, Japan; ^5^ Department of Microbiology, New York University, New York, NY, United States

**Keywords:** galectin-9, COVID-19, SARS-CoV-2, inflammation, lectins

## Abstract

SARS-CoV-2 remains a global health crisis even with effective vaccines and the availability of FDA approved therapies. Efforts to understand the complex disease pathology and develop effective strategies to limit mortality and morbidity are needed. Recent studies reveal circulating Galectin-9 (gal-9), a soluble beta-galactoside binding lectin with immunoregulatory properties, are elevated in SARS-CoV-2 infected individuals with moderate to severe disease. Moreover, *in silico* studies demonstrate gal-9 can potentially competitively bind the ACE2 receptor on susceptible host cells. Here, we determined whether early introduction of exogenous gal-9 following SARS-CoV-2 infection in humanized ACE2 transgenic mice (K18-hACE2) may reduce disease severity. Mice were infected and treated with a single dose of a human recombinant form of gal-9 (rh-gal-9) and monitored for morbidity. Subgroups of mice were humanely euthanized at 2- and 5- days post infection (dpi) for viral levels by plaque assay, immune changes measures by flow cytometry, and soluble mediators by protein analysis from lung tissue and bronchoalveolar Lavage fluid (BALF). Mice treated with rh-gal-9 during acute infection had improved survival compared to PBS treated controls. At 5 dpi, rh-gal-9 treated mice had enhanced viral clearance in the BALF, but not in the lung parenchyma. Increased T and dendritic cells and decreased neutrophil frequencies in the lung at 5 dpi were observed, whereas BALF had elevated levels of type-I interferons and proinflammatory cytokines. These results suggest a role for rh-gal-9 in limiting acute COVID-19. Further studies are required to determine the optimal design of gal-9 treatment to effectively ameliorate COVID-19 disease.

## Introduction

The coronavirus disease 2019 (COVID-19) pandemic, caused by the severe acute respiratory syndrome coronavirus 2 (SARS-CoV-2), has infected over a staggering 360 million individuals and claimed over 20 million lives to date (1, 2). SARS-CoV-2 is an enveloped and single stranded positive-sense RNA virus of the *Coronaviridae* family (3). Unlike other coronaviruses diseases, COVID-19 disease is characterized by a range of clinical presentations from asymptomatic and mild disease with cough, fever, to severe cases of pneumonia and in some cases with vascular injury, cytokine and chemokine storm and death (3). With the advent of vaccines against SARS-CoV-2 infection dissemination, disease severity, and mortality has been dramatically altered, though protection against novel variants of concern and long COVID (post-acute sequalae of COVID-19) are proving an ongoing challenge (4, 5). FDA-approved treatment options targeting both the virus and/or host factors for the various stages and presentations of COVID-19 continue to expand and remain an area of critical need to reduce the risk of hospitalization or death as variants evolve and limit efficacy of many of these agents (6, 7). As COVID-19 persist globally under highly vaccinated populations, the urgency of improved strategies targeting COVID-19 remain an urgent need.

Galectin-9 (gal-9), a tandem-repeat-type lectin with two carbohydrate recognition domains (CRDs) with distinct physiological activity that interacts with β-galactosides (8), is known to be highly immunomodulatory and modulates cell-cell and cell-matrix interactions (8). While gal-9 is shown to have a potential prognostic value in the setting of various cancers and is suggested as a promising target for immunotherapy (9–12), recent studies reveal its significance in SARS-CoV-2 infection and associated sequalae. Individuals with COVID-19 exhibit higher levels of plasma gal-9 compared to healthy controls (13) and the extent of these levels are associated with disease severity and death (14–18). However, structural and sequence alignment comparisons of SARS-CoV-2 receptor binding domain (RBD) and the human galectin-9 N terminal CRD identified fully conserved, highly conserved, and lowly conserved residues between the two (19). Interestingly, gal-9 has been shown to bind to influenza A virus (PR8/H1N1 strain) and block virus attachment to the host cells in a lactose-specific manner (20) as well as restrict hepatitis B virus replication through selective autophagy of viral core proteins *in vitro* (21). Moreover, in a mouse model of influenza, recombinant gal-9 treatment diminished influenza viral replication, reversed weight loss, and reduced blood inflammatory cytokines (20). Hence, introduction of exogenous gal-9 may plays a role in limiting the pathogenesis of several human viral diseases and could prove valuable in the setting of SARS-CoV-2 infection.

Given our understanding of gal-9 modulation in infectious disease models and cancers, the therapeutic potential and *in vivo* effects of gal-9 in SARS-CoV-2 infection and pathogenesis remains largely unexplored. In the present study, we hypothesized that introduction of exogenous gal-9 during the acute phase of infection may decrease SARS-CoV-2 related disease severity. Herein we present our study findings characterizing the effects of early treatment with a human recombinant gal-9 in the SARS-CoV-2 infected K18-hACE2 mouse model.

## Materials and methods

### Viral production and isolation

SARS-CoV-2, isolate USA-WA1/2020 (BEI Resource NR52281, a gift from Dr. Mark Mulligan, New York University Langone Vaccine Center) was amplified once in Vero E6 cells (P1 from the original BEI Stock). Briefly, a 90-95% confluent T175 flask of 1x10^7^ Vero E6 cells was infected with 10µL of the BEI stock in 3 mL of SARS-CoV-2 infection media (DMEM, 2% FBS, 1% NEAA, 10 mM HEPES pH7.0) for 1 hour. After 1 hour, 15 mL of infection media was added to the inoculum and cells were incubated for 3 days at 37°C and 5% CO_2_. After 3 days, the supernatant was collected and the monolayer frozen and thawed once. Both supernatant and cellular fractions were combined, centrifuged at 1200 rpm for 5 min and filtered using a 0.22-µm Steriflip filter unit (Millipore, Burlington, MA, USA)). All experiments with SARS-CoV-2 were performed in the CDC/USDA-approved BSL-3 facility in compliance with the NYU School of Medicine guidelines for Biosafety Level 3.

### Mice

Male C57BL/6 K18-ACE2 mice (6 weeks old) were purchased from Jackson Laboratories (Bar Harbor, ME, USA). All mice used in these studies were between 6 and 8 weeks of age at the start of the experiment. Mice were maintained with food and water ad libitum under a 12-hour dark/light cycle in a pathogen-free facility at New York University Langone Health Center. All experiments were performed with approval by the New York University Langone Health Center Institutional Animal Care and Use Committee and in accordance with guidelines from the National Institutes of Health, the Animal Welfare Act, and the U.S. Federal Law. All ABSL3 procedures was performed in the ABSL3 facility of NYU Grossman School of Medicine (New York, NY), in accordance with its Biosafety Manual and Standard Operating Procedures.

### Cell lines

Vero E6 cells were cultured in DMEM supplemented with 10% FBS and 1% penicillin/streptomycin. Cells were incubated at 37°C in a humidified atmosphere with 5% CO2.

### *In vivo* SARS-CoV-2 infection

For SARS-CoV-2 infection, mice were anesthetized with ketamine/xylazine intraperitoneally and subsequently infected with 1x10^4^ PFU of SARS-CoV-2 in 50µL volume intranasally. In the treatment arm mice received 30µg in 200µL of endotoxin-free PS intraperitoneally 6 hours post SARS-CoV-2 infection.

### *In vitro* SARS-CoV-2 infection and Gal-9 administration

Vero E6 cells were pretreated with or without Gal-9 for 6 hours. Then virus mixture containing indicated concentration of Gal-9 was added to the wells. 24-hour post infection, cells were harvested with TRizol (Thermo Fisher Scientific, 15596026) for RNA extraction or fixed with methanol: acetone (1:1) for IFA assay.

### Plaque assay

SARS-CoV-2 titers were determined by plaque assay on VeroE6 cells. Briefly, 2 x 10^5^ VeroE6 cells/well were seeded in a 24-well plate. The next day, serial 10-fold dilutions of virus, lung lysate, and BAL in DMEM supplemented with 2% FBS were added to the cells and incubated for 1 hour at 37°C. Cells were then overlaid with 0.8% agarose in DMEM + 2% FBS and incubated for 72 hours at 37°C. Cells were fixed with 10% formalin, agarose plug removed, and stained with crystal violet.

### Tissue homogenization

Lungs were harvested at designated time points and washed in 1xPBS before placement in 2mL tubes containing 500µL of sterile 1xPBS and one 5-mm stainless steel bead (QIAGEN). Lung tissue was homogenized with a Retsch MM400 mixer at 30 Hz for 2 min. After homogenization, lung debris was centrifuged at 15,000 rpm for 10 min. Supernatant were transferred to new tubes.

### Bronchoalveolar lavages fluid isolation

Bronchoalveolar lavages (BAL) were obtained by flushing the airway of the mice with 1 mL of saline. Cells and the supernatant were separated by centrifugation. The supernatants were transferred to a new tube.

### Cytokine and chemokine quantification

Lung lysate and BAL supernatant were measured through mouse 23-plex array analysis (Bio-Rad, Hercules, CA, USA) for cytokine and chemokine protein levels and mouse IFN alpha and beta array analysis (Invitrogen, Waltham, MA, USA).

### Flow cytometry

Single-cell suspension of lung tissues were prepared for flow cytometry as previously described (22). Lung tissue were injected and incubated with RPMI 1640 media containing Liberase TM (2mg/mL; Sigma-Aldrich), 10% FBS, 0.2% CaCl_2_, 0.2% MgCl_2_, DNase I (Roche), and 1% HGPG [1mM HEPES, 5mM L-glutamine, penicillin/streptomycin (10,000 U/mL), and gentamicin (5µg/mL) (pH 7.5)] for 30 min at 37°C. Digestion buffer was inactivated by addition of RPMI 1640 media containing 1 mM EDTA and 10% FBS. Lung tissue was dissociated into single-cell suspensions, and tris-buffered ammonium chloride was used to lyse red blood cells. Fc Receptors were blocked with anti-CD16/32 Fc block antibody (Clone: 93, Biolegend) and stained with Live Dead UV (AF350 NHS Ester, ThermoFisher), CD4 – FITC (Clone: GK1.5, Tonbo Biosciences), Ly6G – PerCP-Cy5.5 (Clone: 1A8, Tonbo Biosciences), CD8a – PE-Cy5 (Clone: 53-6.7, Tonbo Bioscience), CD45 – PE-Cy7 (Clone: 30-F11, Tonbo Bioscience), Siglec-F – BV421 (Clone: E50-2440, BD Bioscience), MHCII – V450 (Clone: M5/114.15.2, Tonbo Bioscience), B220 – V500 (Clone: RA3-6B2, Tonbo Bioscience), CD11b – BV711 (Clone: M1/70, Biolegend), Ly6C – BV786 (Clone: HK1.4, Biolegend), CD11c – rF710 (Clone: N418, Tonbo Bioscience) for 20 min. Cells were fixed with 4% paraformaldehyde for 1 hour at room temp and resuspended in FACS buffer. Cell suspension was processed on Bio-Rad ZE5 Yeti instrument, and data analyzed using FlowJo software.

### Plate-based Gal-9/ACE2 binding assay

Enzyme-linked immunosorbent assay (ELISA) was used to evaluate ACE2/Gal-9 interactions. Immuno microplates with 96wells (Thermo Scientific) were coated with either recombinant Human ACE2 (rh-ACE2; 200ng/well; BioLegend) or full-length recombinant human Galectin-9 (rhG9NC; 1µg/well; GalPharma) overnight in 10mM phosphate buffer. Plates were subsequently washed three times (PBS w/0.05% Tween 20) and blocked using a protein-free blocking buffer (Pierce) for 2hrs at room temperature. Plates were then incubated with either rhACE2 or rhG9NC at concentrations indicated. After a 2hr incubation plates were washed and incubated with a secondary antibody towards respective target for an hour; an anti-human ACE2 monoclonal antibody (clone CL4035; ThermoFisher Scientific) or anti-human Galectin-9 monoclonal antibody (clone 9S2-1; BioLegend). For detection, goat anti-mouse IgG (H+L) conjugated to horseradish peroxidase was used for 1 hour. After a final wash, the colorimetric substrate 3,3’,5,5’-Tetramethylbenzidine (ThermoFisher Scientific) was incubated in wells for 20 minutes and the enzymatic reaction was stopped using 0.16M sulfuric acid. Optical density (OD) was measured on at 450nM on a spectrophotometer and absorbance was normalized for background at 650nM correction and OD in control wells. All conditions were measured in duplicate.

### MTT assay

The cytotoxic effect of Gal-9 on Vero E6 cells were detected using MTT assay kit (abcam, ab211091) according to manufacturer’s instructions. In brief, VeroE6 cells were cultured in 96-well plates were incubated with different concentrations of Gal-9. 48-hour post treatment, the media was removed and 100 μl MTT reagent (1:1 dilution in DMEM medium (serum free)) was added to each well and incubated for 3 hour at 37°C. Then the mix was removed, and 150 μl MTT solvent was added into each well. Quantification was performed by reading absorbance at OD=590 nm. The data from three independent experiments was used to calculate the CC50 by nonlinear regression using GraphPad Prism 8.0 software.

### Immunofluorescence

Cells were fixed and permeabilized with methanol: acetone (1:1) for 10 min at 4°C. Then cells were incubated in the 5% goat serum (Seracare Life Sciences Inc, 55600007) at room temperature for 30 min. Next, cells were incubated with the primary antibody (monoclonal rabbit anti-SARS-CoV-2 nucleocapsid antibody (GeneTex, GTX135357) and the secondary antibody (Goat anti-Rabbit IgG (H+L) secondary antibody, FITC (Thermo Fisher, 65-6111)). Finally, cells were incubated with DAPI (300 nM) (Thermo Fisher Scientific, D1306) for 5 min at room temperature. Images were acquired using a fluorescence microscope.

### Image analysis by ImageJ

Single channel images were imported into ImageJ and channels were merged to generate a composite. Brightness/Contrast were corrected based off Mock and Heat Inactivated conditions and applied to all conditions for downstream analysis. To determine the frequency of infected cells, the Cell Counter function was utilized to quantify number of DAPI positive cells and FITC positive cells and frequency of infected cells was determined by FITC+/DAPI+ x 100.

### RT-qPCR

Total RNA was extracted using chloroform-isopropanol-ethanol method according to the instructions. RNA was then reversed transcribed into cDNA using RevertAid First Strand cDNA Synthesis Kit (Thermo Fisher Scientific, K1622) in accordance with the manufacturer’s instructions. RT-PCR was performed for each sample using Taqman Universal Master mix II, with UNG (Thermo Fisher Scientific, 4440038) on a ViiA7 Real time PCR system. Primers and probes for detection of the RNaseP gene and SARS-CoV-2 Nucleocapsid (N) gene were obtained from IDT (2019-nCoV RUO Kit (Integrated DNA Technologies, 10006713)). The expression level of the N gene was determined relative to the endogenous control of the cellular RNaseP gene.

### Statistical analysis

All statistical analysis was performed using Prism 8 GraphPad Software (La Jolla, CA). For experiments comparing 2 groups, two-tail Student t-test was used to determine statistical significance. For survival studies, Log rank Mantel-Cox test was performed. For viral quantification experiments non-Gaussian distribution was assumed and Mann-Whitney test was performed. For experiments comparing three or more groups, one-way ANOVA, Sidak test was used to determine statistical significance for sample groups that assume Gaussian distribution or Kruskal-Wallis H test with sample groups that assume non-Gaussian distribution. Statistical significance is determined as *P<0.05, **P<0.01, ***P<0.001, ****P<0.0001, and N.S. for not significant. Data are represented as mean +/- SEM, unless otherwise specified.

## Results

### Galectin-9 binds to the human ACE2 receptor

**Figure 1:**
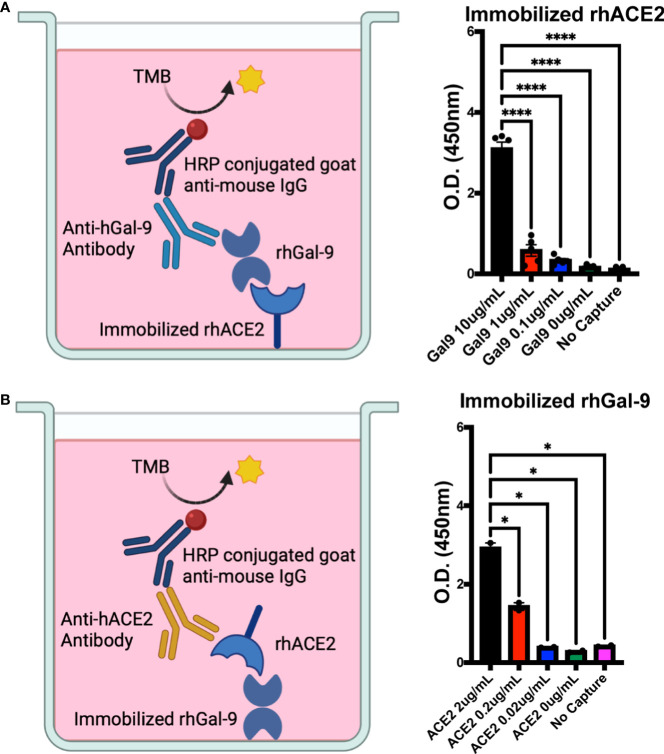
Human galectin-9 binds to the human ACE2 receptor. **(A)** Binding potential of recombinant human galectin-9 (rh-gal-9) to immobilized recombinant human ACE2 (rh-ACE2). **(B)** Binding potential of rh-ACE2 to immobilized rh-gal-9. n = 2-5/group, *p<0.05 and ****p<0.0001. One-Way ANOVA. Data are represented as mean +/- SEM. TMB= chromogenic substrate 3,3’,5,5’-Tetramethylbenzidine; HRP= horseradish peroxidase.

It has recently been shown *in silico* that gal-9 may bind to the ACE2 receptor, which is the canonical SARS-CoV-2 receptor for viral entry into host cells (19). To determine the direct binding potential of gal-9 to the ACE2 receptor, we utilized a plate-based binding assay. We found significant binding of recombinant human galectin-9 (rh-gal-9) to immobilized recombinant human ACE2 (rh-ACE2) in a concentration dependent-manner (**Figure 1A**). This interaction was further validated with soluble rh-ACE2 binding to immobilized rh-gal-9 (**Figure 1B**). These results confer the *in silico* study showing gal-9 feasibility to bind to the ACE2 receptor and potentiate the therapeutic potential of utilizing rh-gal-9 to block the hACE2 receptor.

### Recombinant human galectin-9 decreases SARS-CoV-2 production in VeroE6 cells

**Figure 2:**
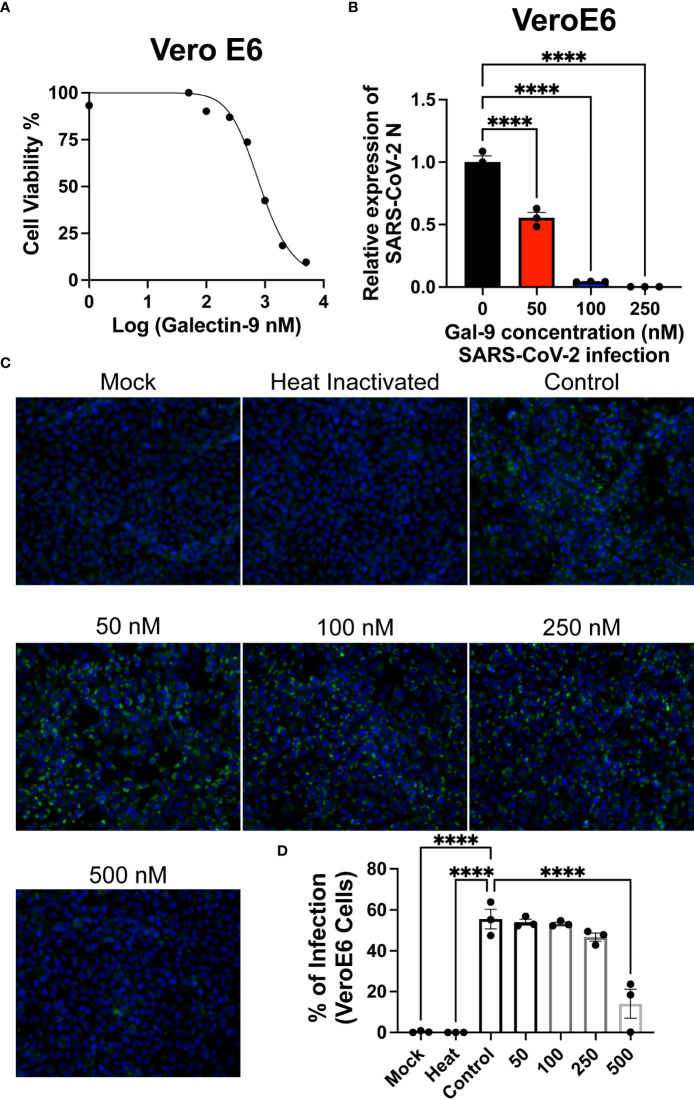
Recombinant human Gal-9 (rh-gal-9) decreases SARS-CoV-2 production in Vero E6 cells. **(A)** Vero E6 cells were treated with different doses of Gal-9 for 48 h followed by measurement of the cytotoxicity of Gal-9 was determined by MTT assay. **(B)** Vero E6 cells were pretreated with Gal-9 at the indicated concentrations for six hours, followed by infection with SARS-CoV-2 (at the MOI of 0.01) for 24 h in the presence of Gal-9. 24 hpi, cells were harvested for RT-qPCR detecting the *N* expression. **(C)** Vero E6 cells were treated with Gal-9 and infected with SARS-CoV-2 as described in **(B)**, cells then were fixed and analyzed by immunofluorescence assay by staining with DAPI (blue) or anti-N Ab (green). **(D)** Quantification of SARS-CoV-2 infected cells in Vero E6 cells (shown in panel C). n=3/group, ****p<0.0001. One-Way ANOVA. Data are represented as mean +/- SEM.

To determine if recombinant human galectin-9 treatment is lethal to cells that express the Ace2 receptor, we treated VeroE6 cells with various concentrations of rh-gal-9 for 48 hours and measured cellular toxicity through MTT assay (**Figure 2A**). We found that with increasing concentration of rh-gal-9 to VeroE6 cells, cellular viability began to diminish at 500 mM concentrations (**Figure 2A**). We next sought to determine if rh-gal-9 could inhibit SARS-CoV-2 infection. To this end, we pretreated VeroE6 cells with or without rh-gal-9 for 6 hours and infected with SARS-CoV-2 for 24 hours and measured SARS-CoV-2 viral production through RT-PCR and found that with increasing concentration of rh-gal-9, the relative expression of SARS-CoV-2 N protein diminishes (**Figure 2B**). Interestingly, when we conducted immunofluorescence on these cells and the SARS-CoV-2 N protein, we did not observe any diminished infection (**Figures 2C, D**). Taken together, these *in vitro* findings suggest that rh-gal-9 is capable of inhibiting SARS-CoV-2 infection in a dose dependent manner.

### Single treatment of recombinant human galectin-9 partially protects against lethal SARS-CoV-2 infection

**Figure 3:**
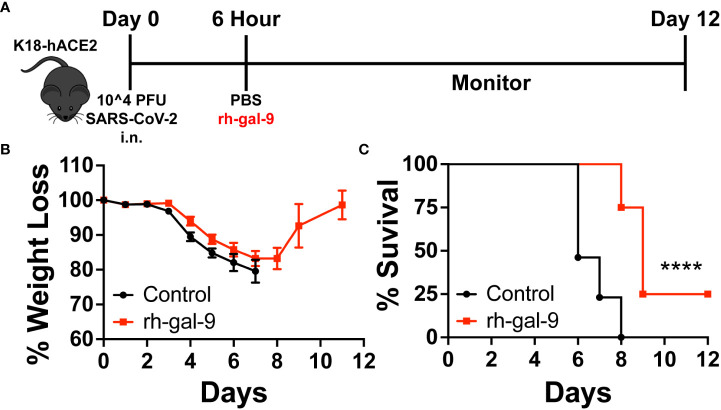
Recombinant human galectin-9 (rh-gal-9) treatment protects against SARS-CoV-2 infection in vivo. **(A)** Schematic for experiment plan for survival studies. **(B)** Weight and **(C)** Survival curve of 6–8-week-old male K18-hACE2 transgenic mice were infected intranasally with 1x10^4 PFU SARS-CoV-2 (USA/WA1/2020), 6 hpi, mice either received a bolus of 1xPBS (black) or 30 µg of rh-gal-9 (red) intraperitoneally. n = 13-23/group, ****p<0.0001. Log-rank (Mantel Cox) test. Data are represented as mean +/- SEM.

Given that rh-gal-9 has potential *in vitro*, to determine the potential of galectin-9 treatment as a therapeutic for SARS-CoV-2 infection *in vivo*, we infected 6–8-week-old K18-hACE2 mice with a lethal dose of SAR-CoV-2 (10^4 PFU) (23, 24). With this known infectious dose, it has been well characterized that K18-hACE2 mice would succumb to infection by 5-7 days post infection (dpi), viral load peaks at 2 dpi in the lung, and gene dysregulation (23, 24). Therefore, using this this dose, we subsequently treated mice with either 30µg rh-gal-9 or PBS intraperitoneally 6 hours post infection to account for the acute infection stage (**Figure 3A**). We found that mice treated with rh-gal-9 exhibited a one-day delay in weight loss initiation compared to PBS control, exhibiting a comparable phenotype to a log-fold less inoculum (**Figure 3B**) (23, 24). Furthermore, we found that in addition to delayed weight loss, mice treated with rh-gal-9 exhibited a partial protection against lethal SARS-CoV-2 infection, characterized by 25% survival rate (**Figure 3C**). Taken together, these data suggest that rh-gal-9 may be an effective treatment for SARS-CoV-2 associated lethality.

### Rh-gal-9 single treatment partially enhanced viral clearance in the alveoli space but not the parenchyma

**Figure 4:**
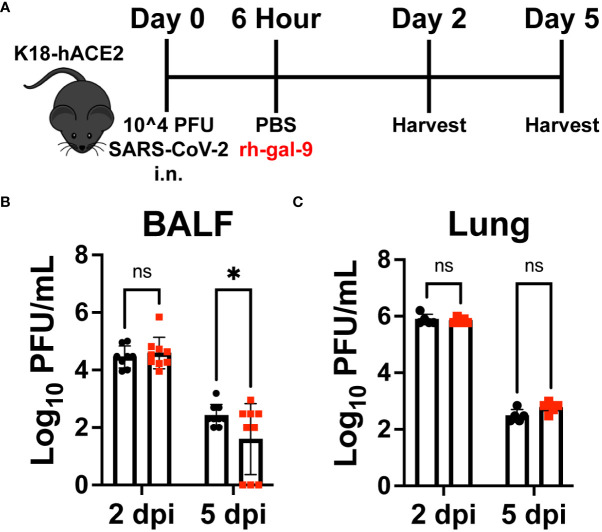
rh-gal-9 treated mice exhibit enhanced viral clearance in the alveoli space but not in the parenchyma. **(A)** Schematic for experiment plan for all timepoint studies. **(B)** Bronchial Alveolar Lavage Fluid (BALF) and **(C)** Lung lysate viral titers by VeroE6 plaque assay at 2- and 5- dpi of PBS (black) or rh-gal-9 (red) treated mice. n = 6-9/group, *p<0.05 and ns, not significant, Two-way ANOVA with Sidak Correction. Data are represented as mean +/- SEM.

Previous work has shown that gal-9 is able to bind to influenza-A and block viral attachment, entry, and replication (20). We therefore next investigated whether early rh-gal-9 treatment is regulating viral replication and/or control resulting in the observed protected phenotype. Additionally, it has also been reported that, gal-9 is capable of restricting hepatitis B virus replication through selective autophagy of viral core proteins *in vitro* (21). To this end, we took whole lung lysate and bronchial alveolar lavage fluid (BALF) of the same animal at 2- and 5- dpi and conducted plaque assays with VeroE6 cells (**Figure 4A**) (25, 26). Surprisingly, we found significant clearance in viral products in the BALF of rh-gal-9 treated mice at 5-dpi (**Figure 4B**). However, we did not observe any difference in viral clearance in lung lysates at 2- or 5- dpi (**Figure 4C**). These data suggest that rh-gal-9 single treatment enhanced viral clearance in the alveoli space but not in the parenchyma in a subset of animals.

### Rh-gal-9 treatment results in altered lung immune response and cytokine profile

**Figure 5:**
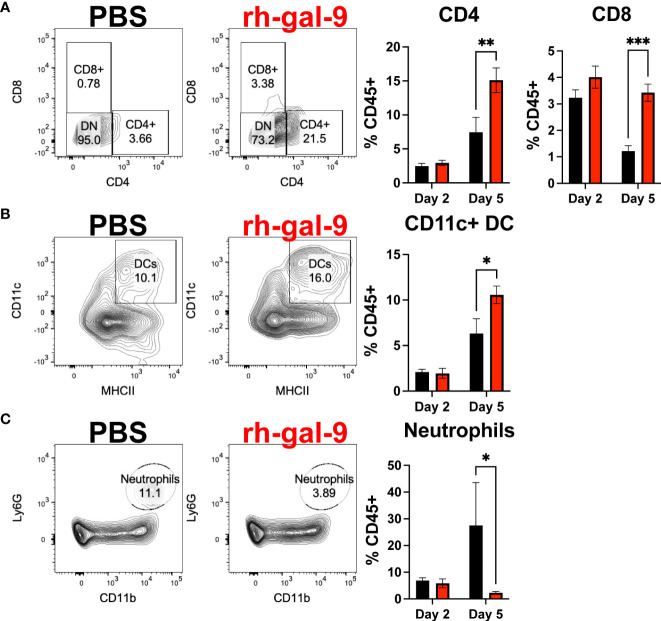
rh-gal-9 treatment increases T cells and CD11c+ DCs and decrease neutrophils at 5 dpi in the Lung. Flow cytometric analysis of **(A)** CD4+ and CD8+ T cells **(B)** CD11c+ DCs, and **(C)** neutrophils at 2- and 5- dpi of PBS (black) or rh-gal-9 (red) treated mice. n=4-5/groups. * p<0.05, ** p<0.01, *** p<0.001, Two-way ANOVA with Sidak Correction. Data are represented as mean +/- SEM.

Several major clinical features of COVID-19 include impaired antiviral cellular responses and increase recruitment of innate immune cells to the lung (27). Therefore, to determine if the protective phenotype observed is a result of an increased antiviral cellular response and/or diminished innate immune cell infiltrate into the lung, we first conducted flow cytometric analysis of the lung at 2- and 5- dpi of PBS or rh-gal-9 treated mice. We found that at 5- dpi, animals treated with rh-gal-9 exhibited increase CD8+ and CD4+ T cells and dendritic cells (DC) in the lung (**Figures 5A, B**). Additionally, we found reduction in neutrophils at 5 dpi (**Figure 5C**). Interestingly, we did not see any differences between groups in B cells and Ly6C+ high monocytes (**Data not shown**). These cellular data suggest that rh-gal-9 treatment promoted a more robust T cell response by blunting the inflammatory cellular milieu.

**Figure 6:**
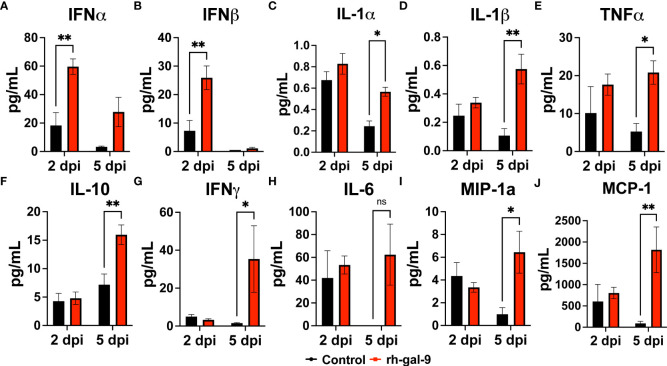
rh-gal-9 treatment increases Type 1 IFN and MyD88 cytokines and chemokines in the BALF. **(A, B)** Type 1 interferon **(A)** IFNγ and **(B)** IFNβ, **(C-H)** MyD88 Cytokines **(C)** IL-1α, **(D)** IL-1β, **(E)** TNFα, **(F)** IL-10, **(G)** IFNγ, **H)** IL-6, and **(I, J)** Chemokines **(I)** MIP-1α and **(J)** MCP-1 at 2- and 5- dpi of PBS (black) or rh-gal-9 (red) treated mice. n=4/group, *p<0.05, and **p<0.01, Two-way ANOVA with Sidak Correction. Data are represented as mean +/- SEM.

Finally, we analyzed soluble factors in the BALF to determine if soluble cytokines and chemokines could be also attribute to the protective phenotype observed in the rh-gal-9 treated animals. We found that compared to PBS, rh-gal-9 treated animals, mice exhibited increased type-I interferon alpha and beta at 2 dpi while no difference was at 5 dpi (**Figures 6A, B** **)**. We also observed significant or trending increase in MyD88 dependent cytokines, such as IL-1α, IL-1β, TNFα, IL-10, IFNγ, IL-6, MIP-1α, and MCP-1 in rh-gal-9 treated animals compared to PBS at 5 dpi (**Figures 6C–J**). Taken together, our data suggests that rh-gal-9 promoted a more robust antiviral response and concurrently promote a delayed inflammatory cytokine response that is not represented by an inflammatory cellular signature.

## Discussion

Success in developing therapies and vaccines against SARS-CoV-2 have dramatically altered the course of the COVID-19 pandemic, however challenges persist. This is compounded by the ongoing evolution of viral variants of unclear pathogenicity, persistent long COVID-19 symptoms, and rebounding infections post-therapy arguing for a need for continued investigation of effective therapies. In the current study, we demonstrated recombinant human galectin-9 is capable of binding to the human ACE2 receptor, a rh-gal-9 concentration dependent inhibition of SARS-CoV-2 infection of VeroE6 cells, and that single treatment with a rh-gal-9 was sufficient to protect K18-hACE2 mice from lethal SARS-CoV-2 infection in the acute phase of disease. This was coupled with enhanced alveolar viral clearance and effector CD4 and CD8 T cell responses in the bronchial alveolar space and lung tissues notable as early as 5 dpi and enhanced type-I interferon production in the lung at 2 dpi. Exogenous gal-9 also resulted in reduced neutrophil recruitment with increase MyD88 dependent cytokine and chemokine responses in the lungs at 5 dpi. Our preclinical evaluation of gal-9 in this SARS-COV-2 infection mouse model provides support for advancing the investigation of harnessing this lectin for patients at various stages of COVID-19 disease.

Previous studies using K18-hACE2 mice have reported comparable trends in viral burden, cellular, and cytokine signatures following lethal SARS-CoV-2 infection (23). While these transgenic mouse studies allowed the evaluation of immunological phenotypes and disease progression with SARS-CoV-2 infection, these predominately have been limited to investigating the overarching phenotype, virological mechanisms, or prophylactic treatments of COVID-19 (23). Therefore, a critical question remaining in the field is whether immunomodulators could be utilized to as a therapeutic for COVID-19. Here, we utilized K18-hACE2 mice that allows for susceptibility of SARS-CoV-2 infection, not feasible in conventional, murine ACE2 expressing, wildtype mice (23, 28, 29). Consistent with previous findings, our data showed that control mice infected with 10^4 PFU intranasal SARS-CoV-2 began to succumb to infection at 6 dpi with 100% mortality at 8 dpi. However, mice treated with rh-gal-9 post-infection exhibited a delay in mortality (8 dpi) and a 25% survival rate. Interestingly, our observed protective phenotype with the rh-gal-9 treatment is comparable to that observed by other groups who have administered multiple treatments of TLR2 inhibitors post infection (30).

Interestingly, in relations to lung and alveolar lavage viral burden, we found reduction in the alveolar lavage at 5 dpi but no change in lung lysates in rh-gal-9 treated K18-hACE2 infected mice. Our data is relatively equivalent to that of viral burdens of rh-gal9 treated animals of influenza infected animals (20). Additionally, consistent to other therapeutics tested in K18-hACE2 mice, viral burdens were reduced in groups that exhibited increase survival frequency compared to controls (31–34).

Importantly, gal-9 is a pleiotropic immune modulator that can alter the innate and adaptive response landscape with SARS-CoV-2 infection. Gal-9 has variable effects on T cells, such as the induction of apoptosis (9), their activation and expansion, and their (35), transendothelial migration (36). While we observed a gal-9 mediated increase in both CD4+ and CD8+ T cells in the lung 5 dpi, this could be an indication of increased infiltration and/or expansion. Interestingly, exogenous gal-9 administration has been previously shown to suppress the presence of neutrophils in the lung in mouse models of emphysema and acute lung injury, similarly to as we demonstrated (37, 38). Of importance as neutrophils are thought to play a significant role in COVID-19 severity (39). Gal-9 treatment is also shown to attenuate acute lung injury in an intranasal LPS inoculation mouse model by expanding plasmacytoid DC-like macrophages in the BALF (38). Considering the effect of gal-9 on pro-inflammatory cytokines and antiviral responses we observed as well, treatment could confer beneficial changes among multiple arms of immunity to improve SARS-CoV-2 outcomes.

Although our data is intriguing, we note several limitations of our study. First, co-immunoprecipitation would have been optimal to elucidate specific ACE2-gal-9 interactions rather than a plate-based binding assay. However, gal-9 interactions are preferentially glycan-mediated, and gal-9 has multiple other known binding partners, both of which may interfere with specific ACE2 interactions. We also observed dissimilarity between the RT-PCR and immunofluorescence results for SARS-CoV-2 presence in Vero E6 cells after Gal-9 stimulation. However, these results confirm Gal-9 treatment limits the production of new viral transcripts but has little to no effect on residual intracellular or cell-bound SARS-CoV-2. Additionally, as our focus of this study was evaluating the immunomodulatory effects of endogenous Gal-9 treatment on SARS-CoV-2 pathogenesis, we did not determine whether this ACE2/gal-9 interaction occurs *in vivo* and has an influence on limiting the entry of SARS-CoV-2 into host cells. Second, the hamster model of SARS-CoV-2 infection is now more commonly used over the humanized ACE2 transgenic mouse model (40). However, while both SARS-CoV-2 infection models are valuable with the inclusion of an immune cell enriched environment as opposed to *in vitro* systems, there is limited information on galectin biology in the hamster model. Finally, other gal-9 mediated mechanisms that were not evaluated in this study may be occurring, including the induction of cell death, cell cycle dysregulation, and downregulation of TLR4 and TLR2 expression (38, 41).

## Conclusion

To conclude, the data presented in this report demonstrates gal-9 as a potential therapeutic in the context of SARS-CoV-2. As we observed an increased survival rate and robust innate and adaptive immune responses with a single administration of gal-9 in SARS-CoV-2 infected K18-hACE2 mice, this opens avenues for studies evaluating multiple dosing and timing strategies to optimize the efficacy of gal-9 treatment. Moreover, our results warrant future studies to replicate these findings in additional models of SARS-CoV-2 infection. While vaccination is the most important defense against SARS-CoV-2, the continued emergence of variants, potentially changing location of pathology in the lung, requires novel treatments resilient against severe COVID-19 outcomes, and we envision gal-9 administration as a promising therapeutic approach.

## Data availability statement

The raw data supporting the conclusions of this article will be made available by the authors, without undue reservation.

## Ethics statement

The animal study was reviewed and approved by New York University Langone Health Center Institutional Animal Care and Use Committee.

## Author contributions

STY, KMK, and LCN designed the study. STY, TAP and LD performed the experiments, TN, KMK, and SKP provided reagents and analytic tools. STY, TAP, LD, KMK, and LCN analyzed the data and wrote the paper.

## Funding

This work was supported by NIH grants R01AI143861 and R01AI143861 Supplement to KMK.

## Acknowledgments

We would like to thank the Office of Science & Research High-Containment Laboratories at NYU Grossman School of Medicine for their support in the completion of this research.

## Conflict of interest

LCN reports grants from the NIH and has received consulting fees from work as a scientific advisor for AbbVie, ViiV Healthcare, and Cytodyn and serves on the Board of Directors of CytoDyn all for work outside of the submitted work. LCN interests were reviewed and are managed by Weill Cornell Medicine in accordance with their conflict-of-interest policies.

The remaining authors declare that the research was conducted in the absence of any commercial or financial relationships that could be construed as a potential conflict of interest.

## Publisher’s note

All claims expressed in this article are solely those of the authors and do not necessarily represent those of their affiliated organizations, or those of the publisher, the editors and the reviewers. Any product that may be evaluated in this article, or claim that may be made by its manufacturer, is not guaranteed or endorsed by the publisher.
